# Transcriptomic signals in blood prior to lung cancer focusing on time to diagnosis and metastasis

**DOI:** 10.1038/s41598-021-86879-8

**Published:** 2021-04-01

**Authors:** Therese H. Nøst, Marit Holden, Tom Dønnem, Hege Bøvelstad, Charlotta Rylander, Eiliv Lund, Torkjel M. Sandanger

**Affiliations:** 1 Department of Community Medicine, UiT - The Arctic University of Norway, Langnes, P.O. Box 6050, 9037 Tromsø, Norway; 2 Norwegian Computing Center, Oslo, Norway; 3 Department of Oncology, University Hospital of Northern Norway, Tromsø, Norway; 4 Department of Clinical Medicine, UiT - The Artic University of Norway, Tromsø, Norway; 5 Department of Child Health and Development, Norwegian Institute of Public Health, Oslo, Norway; 6 Department of Research, Institute of Population-Based Cancer Research, Cancer Registry of Norway, Oslo, Norway

**Keywords:** Biomarkers, Cancer epidemiology, Metastasis

## Abstract

Recent studies have indicated that there are functional genomic signals that can be detected in blood years before cancer diagnosis. This study aimed to assess gene expression in prospective blood samples from the Norwegian Women and Cancer cohort focusing on time to lung cancer diagnosis and metastatic cancer using a nested case–control design. We employed several approaches to statistically analyze the data and the methods indicated that the case–control differences were subtle but most distinguishable in metastatic case–control pairs in the period 0–3 years prior to diagnosis. The genes of interest along with estimated blood cell populations could indicate disruption of immunological processes in blood. The genes identified from approaches focusing on alterations with time to diagnosis were distinct from those focusing on the case–control differences. Our results support that explorative analyses of prospective blood samples could indicate circulating signals of disease-related processes.

## Introduction

Lung cancer is the most commonly diagnosed cancer (2.1 million new cases in 2018) and the leading cause of cancer death worldwide^1^. In Norway, there were about 3200 new cases of lung cancer in 2017 where around half were diagnosed in women^2^. Among women, there is a high proportion of adenocarcinomas^3^; approximately 80% of those diagnosed with known stage have metastasis at diagnosis^2,4^; and survival is related to stage at diagnosis^2^. The majority of these cancers is attributed to exposure to tobacco smoke^5^, and the incidence rate of lung cancer in women in Norway has increased in recent years^2^.

Understanding key molecular markers of lung carcinogenesis and identifying biomarkers for risk stratification and early detection is essential for reducing lung cancer mortality. Several studies have successfully identified clinically relevant biomarkers in tumor or in blood at time of diagnosis, like EGFR mutations or PD-L1 expression^6–11^. Still, as earlier diagnosis is essential for improved prognosis, these markers should ideally be identified in a readily obtainable matrix at a time early in the progression of the malignancy.

Prospective study designs allow for exploration and characterization of early functional genomic events, and recent studies have indicated that there are such molecular signals in blood that can be detected years before cancer diagnosis. As exemplified for breast cancer, modulated trajectories in gene expression linked to breast cancer, especially related to metastatic cancers, have been identified in blood prior to the clinical diagnosis^12,13^. Related to lung cancer, epigenetic markers that largely reflect past and current tobacco smoking have been established as blood-based markers of lung cancer risk using prospective designs^14,15^. Still, little is known regarding peripheral changes in gene expression prior to clinical manifestation of lung cancer^16^.

Gene expression and other ‘omics data’ based on peripheral blood samples are influenced by the underlying distribution of white blood cells at the time of blood sampling, and algorithms can estimate the proportions of such cell populations. Considering that certain cell populations have been linked to elevated cancer risks^17^, estimated proportions of white blood cells in blood as well as their relative ratios could be related to future cancer risk. Indeed, ‘omics data’, exemplified by DNA methylation data in blood, has been used to predict a neutrophil-to-lymphocyte ratio (NLR) that was related to risk of several cancers^18–20^.

This study aimed to identify transcriptomic signals in blood during years prior to lung cancer diagnosis. We also assessed differences in blood cell populations that were estimated from microarray data. To address the prospective design of the study, we employed several approaches to statistically analyze the data with focus on potential signals in samples donated closer to diagnosis and from metastatic cases.

## Methods

### The NOWAC cohort and the study participants

The Norwegian Women and Cancer study (NOWAC) is a nationally representative cohort study initiated in 1991^21^. Women aged 30–70 years were randomly selected from the National Registry and invited to participate in the study through a mailed invitation letter to their home address that also included a detailed questionnaire. Women that agreed to participate have been followed-up regularly with consecutive questionnaires. The questionnaires have covered self-reported anthropometry and lifestyle variables, including detailed information on past and concurrent smoking. Based on the questions related to smoking history, we constructed a comprehensive smoking index (CSI)^22^ as a stringent variable to represent smoking exposure. CSI scores were obtained using duration of smoking (dur; years), intensity (int; average number of cigarettes per day during years of smoking), and time since smoking cessation (tsc; years) and fitting the following model to our data: *X*_2_ = (1 − 0*.*5^dur∗*/τ*^) (0*.*5^tsc∗*/τ*^) ln(int + 1), where τ is the estimated half-life parameter, and δ is an estimated lag time parameter describing tsc and total duration as follows: tsc∗  = max(tsc − δ, 0) and dur∗  = max(dur + tsc − δ) − tsc∗ .

We conducted a case–control study nested within the NOWAC study among those participants who had donated a blood sample in 2003–2006 (N = 48,941)^23^. At the time of blood donation, the participants also filled out a one-page questionnaire covering information about recent and current smoking habits. Blood samples were donated at family doctors into RNA stabilizing PAXgene tubes that were sent by overnight mail to the Department of Community Medicine, UiT, Tromsø, Norway. Upon arrival, tubes were frozen immediately at − 80 °C.

Through linkage with the Cancer Registry of Norway we identified 134 participants who had been diagnosed with lung cancer between 2004 and 2011, after they donated a blood sample. Thus, the time from blood donation to diagnosis ranged from 0.2 to 7.2 years. Of the 134 identified cases, 100 were diagnosed with metastatic cancer at the initial diagnosis. For each case, one cancer-free control was randomly drawn from NOWAC participants with available blood samples and matched on birth year and blood sample collection batch. All participants gave written informed consent and this study was approved by the Regional Committee for Medical and Health Research Ethics in Northern Norway and the Norwegian Data Inspectorate. The research has been conducted according to the principles expressed in the Declaration of Helsinki.

### Microarray analytical methods and data preprocessing

Microarray service was provided by the Genomics Core Facility at the Norwegian University of Science and Technology (NTNU). Briefly, total sample RNA was isolated from the whole blood samples in PAXgene tubes using established protocols^24^. Samples were analyzed using the IlluminaHuman HT-12 expression bead chips and Illumina GenomeStudio 1.9.0 was used to assess the quality of each array. Of the 268 samples analyzed, six case–control pairs were excluded due to laboratory quality measures before original probe values were background corrected using negative controls (R package limma: function nec)^25^. Further, probes reported to have poor quality from Illumina, no annotation or detected in < 10% of samples were removed and values were quantile normalized (R lumi:lumiN) and log2 transformed (R lumi:lumiT)^26^. Annotation of preprocessed data was obtained using R lumi:nuID2RefSeqID and R illuminaHumanv4.db package. The statistical analyses were performed using 11,610 annotated and unique transcripts in 128 case–control pairs.

### Estimations of white blood cell proportions in blood samples

We employed the deconvolution algorithm CIBERSORT^27^ and the LM22 signature matrix to estimate the proportions of 22 white blood cell populations (WBCs) in samples based on the gene expression profiles. Cell types with mean across all samples > 5% for the relative fractions were included in comparisons across case–control status, smoking status and periods of years to diagnosis. An NLR in each sample was calculated based on the fraction of neutrophils to the summed estimated proportions of lymphocyte (details presented in Table S1).

### Data treatment and statistical analyses

Three different approaches were pursued to identify genes that were associated with case–control status, metastatic status and the time interval between blood sampling and the cancer diagnosis.

#### Exploratory methods assessing overall gene expression according to time to diagnosis

Differences in expression values in each case–control pair across time were explored using descriptive and exploratory methods described by Lund et al.^12^ and Holden et al.^13^. The differences according to time were evaluated using both predefined time-dependent alterations (‘curve groups’ method; Lund et al.^12^) and alterations in moving windows in time (‘local in time statistics’ method; Holden et al.^13^). Specifically, the ‘curve group’ method designate genes in predefined groups of genes according to their respective means within three set time windows (days to diagnosis) where the value for each case–control pair is represented by their gene-wise difference in expression values. Six potential curve groups (‘123 =  ’, ‘132 =  ’, ‘312 =  ’, ‘321 =  ’, ‘213 =  ’, ‘231 =  ’) are predefined according to ranked average case–control difference in expression values within the time windows. In the dots provided here for illustration, diagnosis is to the right; Period 1 is closest to diagnosis and the number listed first is the respective period with the highest mean. For example, a gene with increasing differences in expression levels between cases and controls when approaching time of diagnosis would be designated to the curve group ‘123’. In our data, the case–control differences were divided into the three predefined groups according to days to diagnosis for the case. To ensure similar numbers of cases in each time window we defined the following cutoffs: < 1093, 1093–1783, 1783 < days. If there are more genes in a curve group than expected by chance, a time trend is considered present^12^. The analyses were performed separately for metastatic case–control pairs which resulted in 36, 31, 33 case–control pairs in the three set time windows, respectively. From these analyses, unranked lists of genes in the significant curve groups were extracted. The results are not presented for the non-metastatic groups due to the small number of case–control pairs in the three time windows (n = 7, 11, 10, respectively).

The ‘local in time statistics’ (LITS) method uses sliding time windows and the data set was divided into overlapping periods that each contained a set of 30 metastatic cases that were consecutive in time across the time to diagnosis (not performed for non-metastatic groups due to small number of case–control pairs). In this dataset, this division resulted in 71 time periods and each period duration ranged 475–1069 days. We focused on hypothesis testing of case–control differences for cases with metastasis according to time to diagnosis. The null hypothesis of the test was that the expectation for differences in log2 gene expression for the case–control pairs was zero for all genes in all time periods. The null distribution is estimated by permutation of the case and control status in each case–control pair. Detailed method descriptions are presented by Lund et al.^12^ and Holden et al.^13^. From these analyses, p-values from testing mean differences in log2 gene expression for case–control pairs across days from blood sampling until time of diagnosis using LITS are presented.

#### Gene-wise tests examining potential case–control differences in subgroups

Differences in expression values between cases and controls were identified using linear models for microarrays (R limma)^28^. Linear regression models adjusting for the matched pairs were estimated for all subjects as well as restricted to (i) pairs where the case was metastatic, and (ii) metastatic cases sampled within 3 years prior to diagnosis, which is approximately overlapping the period represented by the time window closest to diagnosis in the curve group method described above. From these analyses, separate lists of the 100 genes with the lowest p-values were extracted (‘Top100 lists’). Additionally, separate regression models were estimated for the cumulated cases until each respective year of the interval between blood donation and diagnosis.

#### Gene-wise tests exploring non-linear differences according to time to diagnosis

Non-linear examinations of gene-wise differences in expression levels according to time to diagnosis in cases adjusting for the matched pairs were explored using natural spline regression (3 degrees of freedom) and moderated F-tests for each gene (R limma). The regressions examined the difference in gene expression values for each transcript and each case–control pair (expression value for case minus value for control) across the time window from blood sampling to diagnosis of the case. Analyses were performed for all subjects as well as restricted to (i) metastatic case–control pairs and (ii) metastatic cases sampled within three years prior to diagnosis. From these analyses, separate lists of the 100 genes with the lowest p-values were extracted (‘Top100 lists’).

Gene names, Entrez IDs, and accession numbers for genes in the identified lists from the different analyses were extracted using R packages lumi and org.Hs.eg.db. Lastly, the identified gene lists were compared between approaches mentioned above and functional explorations of molecular signatures as gene ontologies within each list were examined by overrepresentation analyses (R clusterProfiler)^29^.

Stratified analyses by histological subtypes (adenocarcinomas, squamous and small cell carcinomas) were not performed due to low sample sizes.

Case–control differences in proportions of WBCs including the NLRs estimated from gene expression values were assessed using regressions (including analyses restricted to all metastatic cases and the subgroup of metastatic cases sampled less than three years prior to diagnosis). Further, we used regression splines to evaluate any non-linear trends in proportions of WBCs as well as NLR across years to diagnosis.

All statistical analyses were performed using R^30^ and open source packages from R and the Bioconductor project. Wilcoxon and Kruskal–Wallis tests were used to test for group differences.

## Results

### Characteristics of study participants

Characteristics of the NOWAC participants are summarized in Table 1. There were more current smokers at blood sampling among cases (63%) as compared to among controls (32%). Table 2 presents the distribution of cases according to case, metastasis, histological subtype and smoking status across years between blood sample donation and cancer diagnosis.

**Table 1:** Characteristics of the NOWAC participants.

	Cases n = 128		Controls n = 128	
	Mean	SD	Mean	SD
**Participant characteristics**				
Age at blood sampling	56.6	4.0	56.6	4.0
Packyears	20.8	11.1	14.3	10.2
CSI	1.4	0.7	0.7	0.8
Age at diagnosis	60.5	4.2		
Time to diagnosis	4.3	2.0		

	n	%	n	%
**Smoking status**				
Current	80	62.5	41	32.0
Former	35	27.3	36	28.1
Never	13	10.2	51	39.8

	n	%		
**Histological subtypes**				
Adenocarcinomas	62	48.4		
Small cell carcinomas	25	19.5		
Squamous cell carcinomas	19	14.8		
Others	22	17.2		

*SD* standard deviation, *CSI* Cumulative smoking intensity.

**Table 2:** Number of cases according to years to diagnosis for all cases, metastatic cases, histological subtypes and smoking status.

Variable\years to diagnosis	0–1	> 1–2	> 2–3	> 3–4	> 4–5	> 5–6	> 6–7	> 7–8	Sum
All cases	15	17	11	18	25	26	10	6	128
Metastatic cases	12	14	10	11	21	19	7	6	100
**Histological subtypes**									
Adenocarcinoma	5	7	5	10	12	16	4	3	62
Squamous or small cell	5	6	5	7	9	6	4	2	44
Other	5	4	1	1	4	4	2	1	22
**Smoking status**									
Current smoker cases	10	12	8	11	15	16	4	4	80
Former smoker cases	5	3	2	5	7	8	4	1	35
Never smoker cases	0	2	1	2	3	2	2	1	13

### Signals in estimated white blood cell compositions

The most prevalent populations of WBCs were neutrophils, CD8 T cells, and monocytes (Table 3). The proportions of neutrophils as well as NLRs were higher in cases as compared to controls (Fig. S1, Table 3). The NLR was also higher in metastatic cases as compared to their matched controls (Table S1) and the case–control difference was more pronounced when further restricted to metastatic cases and controls within 3 years of the diagnosis. Still, it appeared that the change was occurring among controls rather than cases. The proportions of resting NK cells were lower in current smokers as compared to former or never smokers (Fig. S2, Table 3) and the mean NLR was similar across smoking categories (Table S1). Assessed in cases only, there were differences in proportions of monocytes across years to diagnosis as assessed by regression splines (Fig. S3, Table S2), but also among activated NK cells and macrophages M0.

**Table 3:** Summary statistics for the estimated proportions of white blood cell populations in the samples with p-values for tests of differences according to case–control status and smoking status.

Cell population	Median cases	Median controls	Case–control pairs			Smoking status
			p-value All^a^	p-value Meta^b^	p-value Meta < 3 years^c^	p-value^d^
Neutrophils	0.24	0.22	**0.04**	**0.04**	**0.05**	0.12
T cells CD8	0.18	0.2	0.48	0.58	0.12	0.38
Monocytes	0.18	0.18	0.91	0.86	0.17	0.65
T cells regulatory	0.12	0.12	0.4	0.28	0.38	0.81
T cells CD4 naive	0.09	0.09	1.00	0.65	0.90	0.28
NK cells activated	0.06	0.06	0.07	0.14	0.27	0.09
NK cells resting	0.06	0.06	0.89	0.84	0.43	**0.01**
T cells gamma delta	0.02	0.02	0.66	0.77	0.92	0.59
B cells memory	0.02	0.01	0.19	0.09	0.65	0.09
Macrophages M0	0.01	0.01	0.10	**0.04**	**0.001**	0.07
T cells CD4 memory activated	0.01	0.01	0.19	0.34	0.24	0.38
Mast cells resting	0.01	0.01	0.13	0.54	0.99	0.15
Neutrophils-to-lymphocytes ratios	0.65	0.55	**0.04**	0.08	**0.01**	0.05

Median values for cases and controls in the subgroups analyzed and in smoking status groups are presented in Table S1.

B cells naïve, dendritic cells activated, dendritic cells resting, eosinophils, macrophages M1, macrophages M2, mast cells activated, plasma cells, T cells CD4 memory resting, T cells follicular helper were estimated as not present in the blood samples.

^a^p-value for Wilcoxon Rank Sum group test including all case control pairs (n = 128 pairs).

^b^p-value for Wilcoxon Rank Sum group test including metastatic case control pairs (n = 100 pairs).

^c^p-value for Wilcoxon Rank Sum group test including all case control pairs (n = 36 pairs).

^d^p-value for Kruskal–Wallis group test including current (n = 121), former (n = 71) and never (n = 64) smokers.

Bold values indicate values below 0.05

### Exploratory assessments of signals across time for metastatic cases

**Figure 1:**
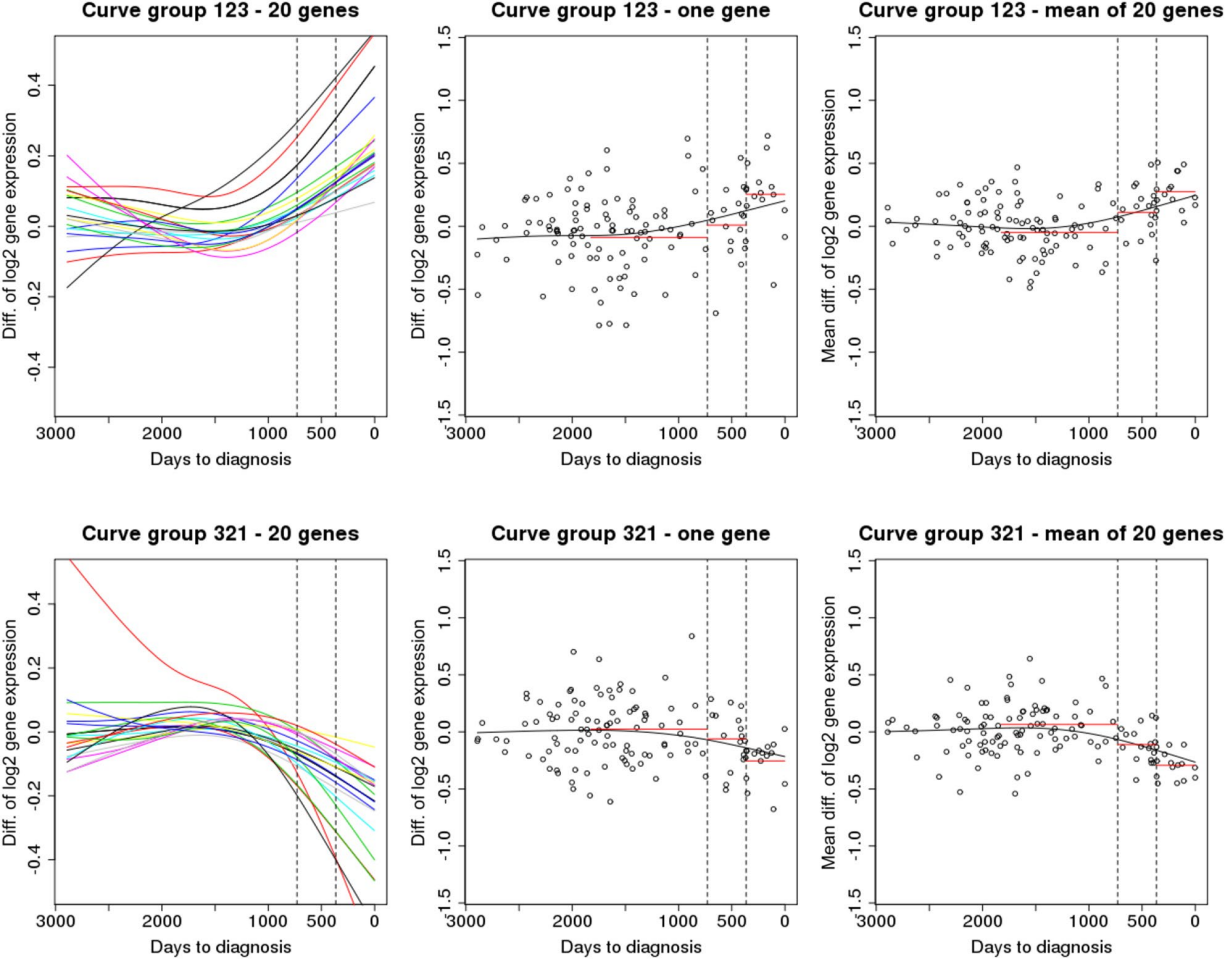
Curve group ‘123’ (upper panels; differences in gene expression values for cases and matched controls highest closest to diagnosis) and ‘321’ (lower panels; gene expression differences highest further from diagnosis) according to time to diagnosis. The left panels present spline-estimated curves for 20 randomly selected genes from each given curve group for illustrational purposes. The dashed, vertical lines indicate the last 2 years prior to diagnosis. In the middle panel the circles represent the differences in gene expression for each case–control pair for one randomly chosen gene, and the respective mean values in each time period are displayed in red and a spline-estimated curve with the gene expression differences for the same gene is displayed in black. The right panels display the same results as the middle panel but calculated for 20 genes.

Analyses of curve groups demonstrated that there were time trends for certain groups of genes in cases with metastatic cancer (p = 0.04; Table 4). Table 4 displays the number of observed and expected genes in each curve group. When testing whether the differences within curve groups differed across time windows, it appeared that the differences were larger in time window closest to the diagnosis (Period 1; Table 4). An illustration of the curve groups that appeared significant, ‘123’ and ‘321’, is presented in Fig. 1.

**Table 4:** The number of genes identified in curve groups for metastatic cases (n = 33, 31, 36 in the three time windows, respectively) and the associated p-values for testing curve groups overall and for each curve group separately in each time window.

Curve group	No. of genes (expected no of genes)	p-values			
		Each curve group overall	Period 3	Period 2	Period 1
Global	4880 (2606)	**0.04**			
123	654 (430)	0.17	0.33	0.29	**0.02**
132	886 (438)	0.10	0.74	0.46	0.40
312	873 (439)	0.10	0.38	0.53	0.37
321	465 (422)	0.28	0.30	0.22	**0.01**
231	969 (440)	0.08	0.22	0.81	0.21
213	1033 (436)	0.06	0.11	0.69	0.48

Period 1 is closest to diagnosis and the number listed first is the respective period with the highest mean. In the dots provided for illustration, diagnosis is to the right.

Bold values indicate values below 0.05

Local in time statistics analyses of differences in log_2_ gene expression for metastatic case–control pairs with time to diagnosis demonstrated that p-values were lowest, although not significant, closest to diagnosis (in the days 1–651 before diagnosis; Fig. S4) but also in another period around 2000 days before diagnosis. Based on results of the curve group and local in time statistics methods, it appeared that for some genes, the distribution of differences in log2 normal values for each case–control pair was dependent on time to diagnosis.

### Gene-wise examinations of non-linear trends according to time to diagnosis

No associations were found for case–control differences in gene expression according to time to diagnosis when explored using spline regression of all case–control pairs (Table S3) or in analyses restricted to metastatic case–control pairs (Table S4). The gene with the lowest p-value for a non-linear trend in the absolute case–control differences in gene expression according to time to diagnosis in cases was *FGFR3* (Fig. S5), which was also among the genes with the lowest p-values when restricting the analyses to cases with metastasis and their matched controls. The magnitude and direction of the spline coefficients were not consistent among the genes with the lowest p-values.

### Gene-wise assessments of differential expression among cases and contro*ls*

Considering a false discovery rate of 0.05, we identified no differences in gene expression between cases and their matched controls in the total study sample (n = 256, Table S5), or when restricting the sample to metastatic cases and their matched control (n = 200, Table S6). However, when further restricting the analyses to metastatic cases within the last three years prior to diagnosis (similar time period as that in the first curve group window), 27 genes were differentially expressed between cases and their matched controls (Table S7). Among the Top100 genes in the analyses including the total study sample, 65 were overlapping with the Top100 genes identified for the metastatic case-controls. Among the Top100 genes in the analyses including the total study sample, 13 were overlapping with the 27 genes differentially expressed in the metastatic case–control pairs sampled within the last three years prior to diagnosis. The 100 genes with the lowest p-values in the analyses using all cases and controls (n = 256) are presented in Table S5. The corresponding list of genes for metastatic cases and paired controls and metastatic cases and paired controls sampled under three years before diagnosis are presented in Tables S6 and S7. The top four genes identified for case–control differences in the total study sample (*TREM1*, *FGFR3*, *MUC1*, *LRRN3*; Table S5) were assigned to curve groups 123, 213, 321, 213, respectively.

When analyzing all metastatic case–control pairs in subsets of accumulated cases 0–1 years, 0–2 years and 0–3 years prior to diagnosis, zero, 61, and 27 genes were differentially expressed (FDR ≤ 0.05), respectively (Table 5). Among the genes identified for years 2 and 3, 20 genes overlapped (indicated in Table S7) and 14 of these were overexpressed in cases. Corresponding analyses are not presented for non-metastatic cancers (n = 28 pairs) as the number of samples in each year were small.

**Table 5:** Number of significant genes identified in analyses of metastatic cases according to years between blood sampling and diagnosis.

Year	No. of cases (cumulated)	FDR 0.05	FDR 0.1
< 1	12	0	42
< 2	26	61	279
< 3	36	27	128
< 4	47	0	0
< 5	68	0	0
< 6	87	0	0
< 7	94	0	1
< 8	100	0	0

When adjusting the regression analysis including all case–control pairs for CSI scores, four of the five genes with the lowest p-values were overlapping with the unadjusted results and test statistics were similar as in those from the unadjusted regressions (results not presented). For the hundred genes with lowest p-values in the CSI-adjusted analyses, the overlap with the Top100 lists for the unadjusted analyses of all pairs, metastatic pairs, and metastatic pairs with < 3 years to diagnosis was 76, 58, and 23, respectively.

### Comparing the identified genes related to time of lung cancer diagnosis and metastatic cancer

**Figure 2:**
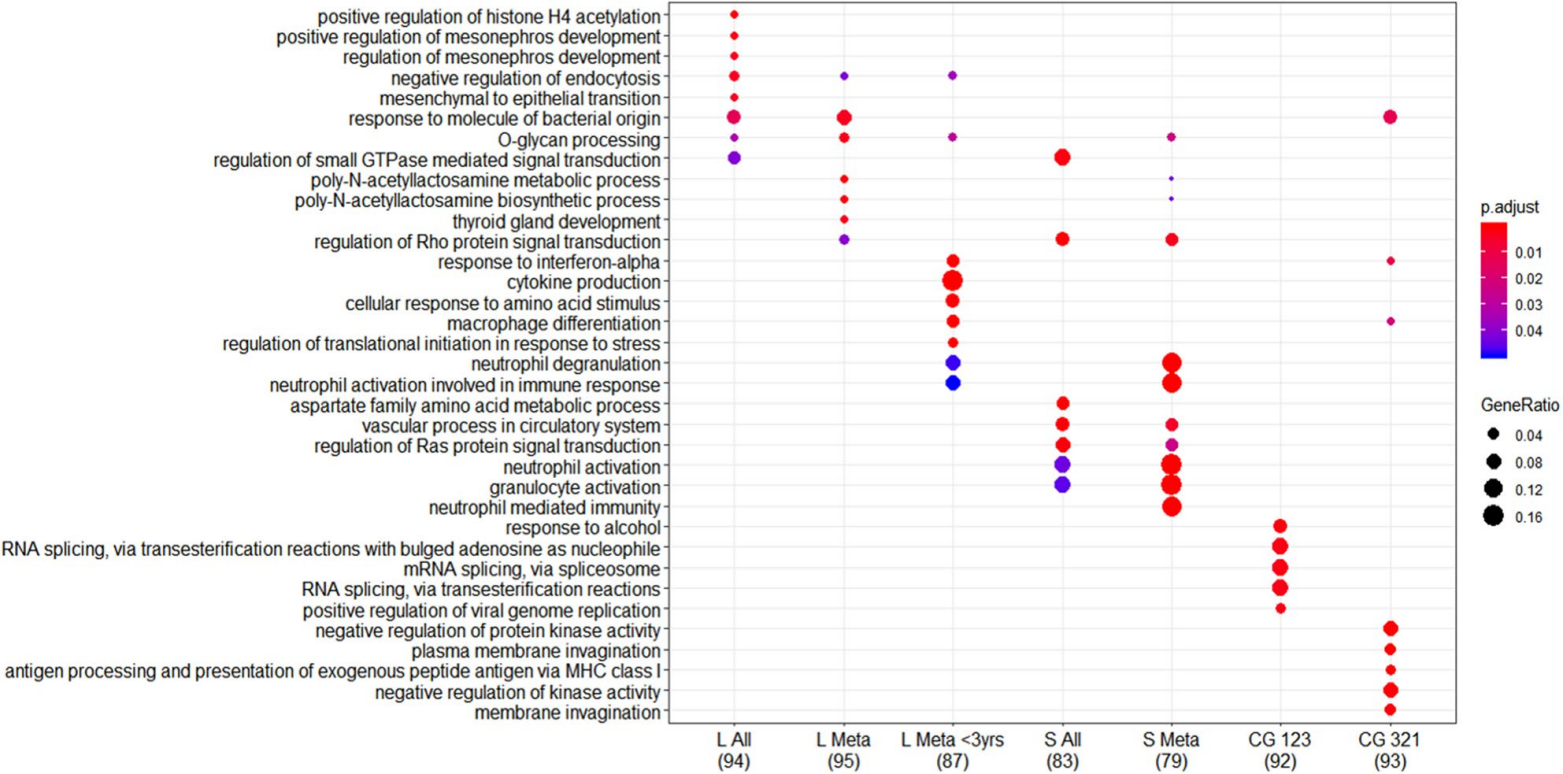
Visualizations of biological processes within gene ontology categories for top lists of interesting genes indicated in the different approaches described in the results. The color scale indicates significance of the overrepresentation of genes and the gene ratio signifies the number of genes in each list relative to the number of genes in ontology categories. L designate the models examined using linear regression in the ‘limma’ package (‘L All’; n = 128 pairs); all pairs with metastatic cases (‘L Meta’, n = 100 pairs); and all pairs with metastatic cases sampled within three years prior to diagnosis (‘L Meta < 3 years’; n = 36 pairs), whereas S designate the spline regressions examined for all pairs and all pairs with metastatic cases using ‘limma’. For the significant curve groups identified, CG 123 and CG 321, the genes in the two significant curve groups were presented. Numbers in brackets signify the numbers of genes in each list with identified Entrez IDs that were included into the overrepresentation analyses. Graphical representation of ontologies were generated using the R package clusterProfiler^29^.

We identified seven lists of genes of interest: Top100 genes for case–control differences in the total study sample, the corresponding Top100 genes for metastatic case–control pairs, and the Top100 genes for metastatic case–control pairs in the last 3 years before diagnosis; the Top100 genes for non-linear associations with time to diagnosis in all cases and the corresponding Top100 genes for metastatic cases; and genes overrepresented in curve groups 123 and 321. For these lists, the overrepresentation analyses of gene ontology categories demonstrated differences in categories among the different approaches (Fig. 2, Table S8) and included metabolic processes and cellular responses to stress (Fig. 2).

## Discussion

### Main findings

These exploratory analyses of microarray gene expression data in peripheral blood focusing on time-dependent processes in lung cancer cases and their matched controls indicated small differences in gene expression during the years prior to diagnosis. Overall, the methods indicated that case–control differences were most distinguishable in metastatic case–control pairs in the period 0–3 years prior to diagnosis. The genes and gene ontology categories identified from approaches focusing on alterations with time to diagnosis were distinct from those focusing on the case–control differences. The enriched processes included categories related to both metabolic processes and immunological responses. White blood cell populations in blood estimated from the gene expression values indicated that neutrophils and NLR might be disrupted in cases overall (independent of time to diagnosis) and that natural killer cells were lower in current smokers. Only few of the genes identified were among those that have been previously linked to tobacco exposure. Overall, the results could indicate disruption of immunological processes in blood of lung cancer cases and that there is a relation to time to diagnosis and metastatic cancer.

### Exploring alterations with time to diagnosis

Of the different curve groups, the case–control differences were increasing and decreasing with time towards diagnosis in the curve groups ‘123’ and ‘321’, respectively, and both gene groups indicated that the period closest to diagnosis (0–2 years) was when case–control differences diverged the most. The LITS method indicated that the gene expression for cases and controls were most dissimilar in the periods around 2.5 in addition to around 4.6 years prior to diagnosis. The non-linear regressions could have indicated diverging trajectories, but this approach did not indicate a specific period where case control differences were more pronounced nor were the direction of effect estimates consistent across top genes. When assessing differentially expressed genes when comparing cases and matched controls while restricting the analysis to cases diagnosed with metastatic cancers within two and three years of diagnosis, 61 and 27 genes were indicated, respectively (20 overlapped). Overall, case–control differences were more pronounced for metastatic cases and controls across the statistical approaches. When comparing all case–control pairs, no significant genes were indicated according to FDR adjusted p-values. In summary, exploring alterations with time in gene expression demonstrated that the magnitude of differences vary according to time to diagnosis.

Summarizing the different approaches, the period closest to diagnosis (approximately 0–3 years prior to diagnosis) was indicated as the period with the more pronounced case–control differences. This period could be expected based on knowledge of the progression of lung cancer disease. The lung cancer tumor grows for several years^31^ and lung cancer diagnosis is preceded by many symptoms especially in the last year before diagnosis is set^32^. A large Danish population-based study investigating trajectories across individuals prior to cancer diagnosis found that lung cancer had up to 30 prior disorders and that most of them accumulated 1–2 years prior to the cancer diagnosis^33^. Thus, the detected alterations could be systemic signals related to lung cancer progression per se, or to any of the related diseases. The ontology categories identified for the included lists indicated several cellular processes, including categories related to both metabolic processes and immunological responses. These could indicate that the systemic signatures of exposure or disease-related processes are linked to these cellular processes in circulating white blood cells.

### White blood cell composition

The proportions of macrophages were higher in metastatic cases as compared to controls and the differences were more pronounced closer to diagnosis. Macrophages have been shown to be tumor promoting and present in tumors^34^. Further, proportions of monocytes increased in all cases closer to diagnosis and such cells in blood samples have been related to lung cancer and presence of late stage disease^35^. Still, the systemic presence and function of macrophages and monocytes prior to clinical manifestation of the disease is not clear. Although proportions of natural killer cells were not different across case–control comparisons, this cell type was associated with exposure to smoking as estimated proportions of natural killer cells were lower in current smokers, which is also observed from cell counts in blood^36,37^. Of note, the differences in natural killer cells across smoking categories here correspond to the observation of estimated fractions based on DNA methylation in the same samples^38^.

Elevated systemic inflammatory responses are important indicators of cancer development and progression^39,40^ and several immune cells in peripheral blood have been shown to have prognostic value for several cancers^17^. The estimated proportions of neutrophils constituted a large fraction of WBCs in samples but was lower as estimated from gene expression than what is typical in blood^41,42^ as well as estimated from DNA methylation in the same samples^38^. Still, their proportions were higher in cases as compared to controls although there was no indication of enhanced differences among metastatic case–control pairs nor with time to diagnosis. The role of neutrophils in carcinogenesis is both pro-tumor and anti-tumor but it is also plausible that their increased presence in blood is a secondary inflammatory response to the carcinogenesis^41–43^.

Related to the elevated proportions of neutrophils in cases as compared to their controls, the estimated NLRs were higher in cases. A higher NLR ratio in blood has previously been associated with poorer survival in lung cancer cases and thus an elevated ratio in blood samples from prospective cases could also be expected^35,42^. Further, NLRs derived from ‘omics data’ has indicated that an elevated NLR (estimated from DNA methylation data) was linked to increased risk of future lung cancer^19^ and lower lung cancer survival^18,20^. Thus, these observations support that there are systemic immunological responses detectable in blood prior to clinical cancer diagnosis, and that using ‘omics data’ as digital cytometry can be useful as predictive markers.

### Indicated genes of interest

The different statistical approaches yielded different lists of top genes with variable degree of overlap. The genes identified from approaches focusing on time to diagnosis and metastatic cancer were distinct from those focusing on the case–control differences in all pairs. Among the 20 genes differentially expressed in blood sampled within both 2 or 3 years prior to diagnosis in metastatic cases, *F5*, *TLR5* and *C19orf59* have been previously observed differently expressed in whole blood of non-small cell lung cancer (NSCLC) patients compared to controls^44^ in one study and *SLC25A5* in another study of blood samples from NSCLC cases and controls^45^. None of the 20 genes were among those 29 or 8 genes that were identified in blood drawn at diagnosis from patients with NSCLC^46^ or adenocarcinomas^47^, respectively. The 20 genes of highest interest in these prospectively sampled blood samples could indicate a specific gene profile in blood prior to lung cancer diagnosis, which would thus be different from those genes identified at diagnosis or later in the disease progression. Accordingly, the *FGFR3* gene, a gene that has been linked to lung carcinogenesis and identified as a therapeutic target^11,48^, was the gene with the lowest p-values in the spline regressions according to time to diagnosis but not when comparing case–control differences. Conversely, these 20 genes could also indicate a non-specific blood profile indicating systemic responses to any cancer developing. Still, the 20 genes indicated here were not among those 50 indicated in a profile related to later breast cancer in a similar prospective case–control study related to breast cancer, also within the NOWAC cohort^24^. Of note, genes of interest identified in blood and in matched tumor samples from the same persons has been compared for women with breast cancer in the NOWAC cohort and the biological processes and expression patterns appeared to vary^49^. The processes indicated in tumor samples were enriched for genes involved in hallmarks of cancer, while processes indicated in blood samples (sampled with the same protocol as this study) were enriched for either general cellular processes or specific immune responses. Thus, circulating profiles of samples in studies related to cancer appear to differ between cancer sites and differ from local molecular signals related to the developing tumor. Further assessments focusing on time to diagnosis and cancer stage are warranted to assess whether disruption of expression of specific genes identified in prospective studies could contribute to risk stratification, diagnostic characterization or indicate genes as therapeutic targets.

To better explore whether the identified genes were linked to exposure to smoking we compared our genes of interest to a large previous meta-analyses of gene expression in 10,233 participants^50^. Three genes (*MUC1, LRRN3, EIF1*) among the ten genes with the lowest p-values in the comparisons of all case–control pairs were among those 1270 differently expressed genes observed when comparing current and never smokers in the meta-analyses. Further, among the hundred with the lowest p-values in the case–control comparisons in this study, fourteen (*MGAT3, KCNMB1, ITGAX, ATP1B1, WDR61, PPP1R14B, ADAM23, NCF4, ALDH1A1, PDCD2, UQCRFS1, MAPRE2, AB11FIP1, GFRA2*) were also identified in the meta-analyses. When investigating metastatic cases under three years, none of the top ten were among the list linked to current smoking, but ten (*MUC1, GSK3B, CD247, ASGR2, PYHIN1, NCF4, GK5, FAM43A, CYP1B1, ID2*) of those 100 with the lowest p-values were linked to smoking in the meta-analyses^50^. Thus, the genes identified linked to lung cancer case–control status, especially when focusing on short time to diagnosis and metastatic cancer, have not been strongly linked to smoking in other populations.

The ontology categories indicated from each statistical approach differed considerably. Many ontology categories with the highest number of genes overrepresented indicated immunological functions and were identified from the lists from linear regressions of metastatic cases < 3 years prior to diagnosis and the spline regressions of all and metastatic case–control pairs. Both the estimated proportions of neutrophils, macrophages and monocytes as well as the ontology categories from genes of interest indicate disruption of immunological processes in blood and that there is a relation to time to diagnosis and metastatic disease for the magnitude of differences. Using blood ‘omics data’ to reveal characteristics of the immune system has been highlighted as part of the development of diagnostic biomarkers and personalized treatment options^51^. Thus, genes identified in exploratory studies of blood transcriptomes could signify systemic signals of local diseases but as gene expression in blood samples to a large extend is influenced by white blood cells it is likely that signals reflect systemic immune responses. Further, blood transcriptome profiles have been shown to distinguish between several pulmonary diseases^44^ and as the disease entails accumulation of many disorders in the time close to clinical diagnosis^33^, signals as those in this study can represent interesting circulating markers during the development of lung cancer.

### The approaches chosen

This study used descriptive, exploratory methods as well as common statistical approaches to explore how differences in gene expression in case–control pairs vary across time between blood sampling and lung cancer diagnosis. By focusing on the time aspects and metastatic cancers while considering the matched pairs, this study might have captured genes of interest that were not apparent from the methods focusing solely on the case–control differences and thus have indicated genes related to metastatic disease and dynamic processes. Further, the signals could indicate a period where perturbations start and indicate functional processes disturbed in blood. The curve group approach included hypothesis testing of curve trajectories while the LITS method is more flexible as it does not assume predefined trajectories. Adjustment of smoking was not feasible in the explorative methods, but when the baseline linear model was adjusted for CSI scores, the overall results did not change substantially. Using established methods in combination with new statistical methods, this study demonstrated subtle time-dependent changes in gene expression profiles in blood prior to clinical diagnosis.

Notably, the prospective case–control design of this study only allowed for investigations of changes in gene expression according to time to diagnoses that are common *across* different individuals. I.e. we assume divergence in gene expression for different persons across time to diagnosis and is not a longitudinal sample although it was analyzed as such. Additionally, the interpretation of study findings are further hampered by the limited sample size of the study. Finally, the blood samples represent snapshots of the circulating immune cell activity and should be interpreted as such.

## Conclusions

Combining approaches focusing on time to diagnosis and metastatic disease revealed distinct signals related to these features and the results could reflect systemic immune responses or disturbed distributions of blood immune cells. These results supported that genes of interest indicated in explorative analyses of prospective blood samples could potentially be linked to systemic signals of disease-related processes.

## Supplementary Information

Supplementary Figures.

Supplementary Tables.

## Data Availability

The microarray data generated and/or analyzed in the current study could be accessed upon reasonable request to the originating cohort. Access will be conditional to adherence to local ethical and security policy. R codes used for the analyses presented in the paper are available upon request.
