# Supplementary material for: Transcriptomic signals in blood prior to lung cancer focusing on time to diagnosis and metastasis
Source: Sci Rep. 2021 Apr 1;11:7406. doi: 10.1038/s41598-021-86879-8 (PMC8017014; doi:10.1038/s41598-021-86879-8)
Supplement: Supplementary file 1 — Supplementary Figures. [file 41598_2021_86879_MOESM1_ESM.docx]

**Supplementary information for**

**Transcriptomic signals in blood prior to lung cancer focusing on time to diagnosis and metastasis**

Therese H Nøst^1*^, Marit Holden^2^, Tom Dønnem^3,4^, Hege Bøvelstad^5^, Charlotta Rylander^1^, Eiliv Lund^1,6^, Torkjel M Sandanger^1^

^1^ Department of Community Medicine, UiT - the Arctic University of Norway, Tromsø, Norway;

^2^ Norwegian Computing Center, Oslo, Norway;

^3^ Department of Oncology, University Hospital of Northern Norway, Tromsø, Norway;

^4^ Department of Clinical Medicine, UiT - the Artic University of Norway, Tromsø, Norway;

^5^ Department of Child Health and Development, Norwegian Institute of Public Health, Oslo, Norway;

^6^ Department of Research, Institute of Population-Based Cancer Research, Cancer Registry of Norway, Oslo, Norway.

**Corresponding author**: Therese Haugdahl Nøst, UiT - the Arctic University of Norway, P.O. Box 6050 Langnes, NO-9037 Tromsø, Norway; E-mail: [therese.h.nost@uit.no](mailto:therese.h.nost@uit.no)

**
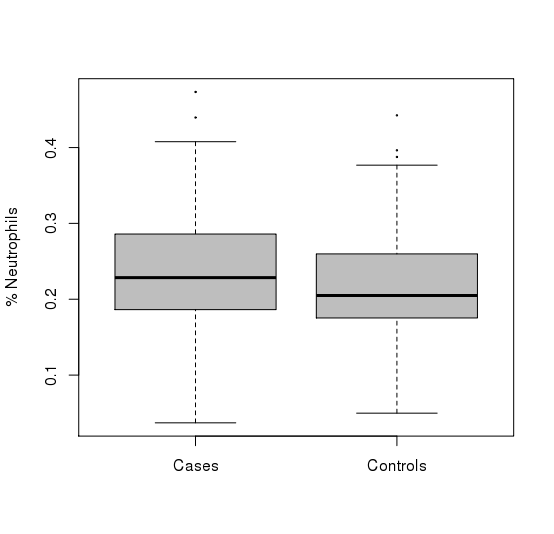
**

**Figure S1: Boxplot of estimated proportion of neutrophils in blood samples obtained from cases (n=128) and controls (n=128).**

**
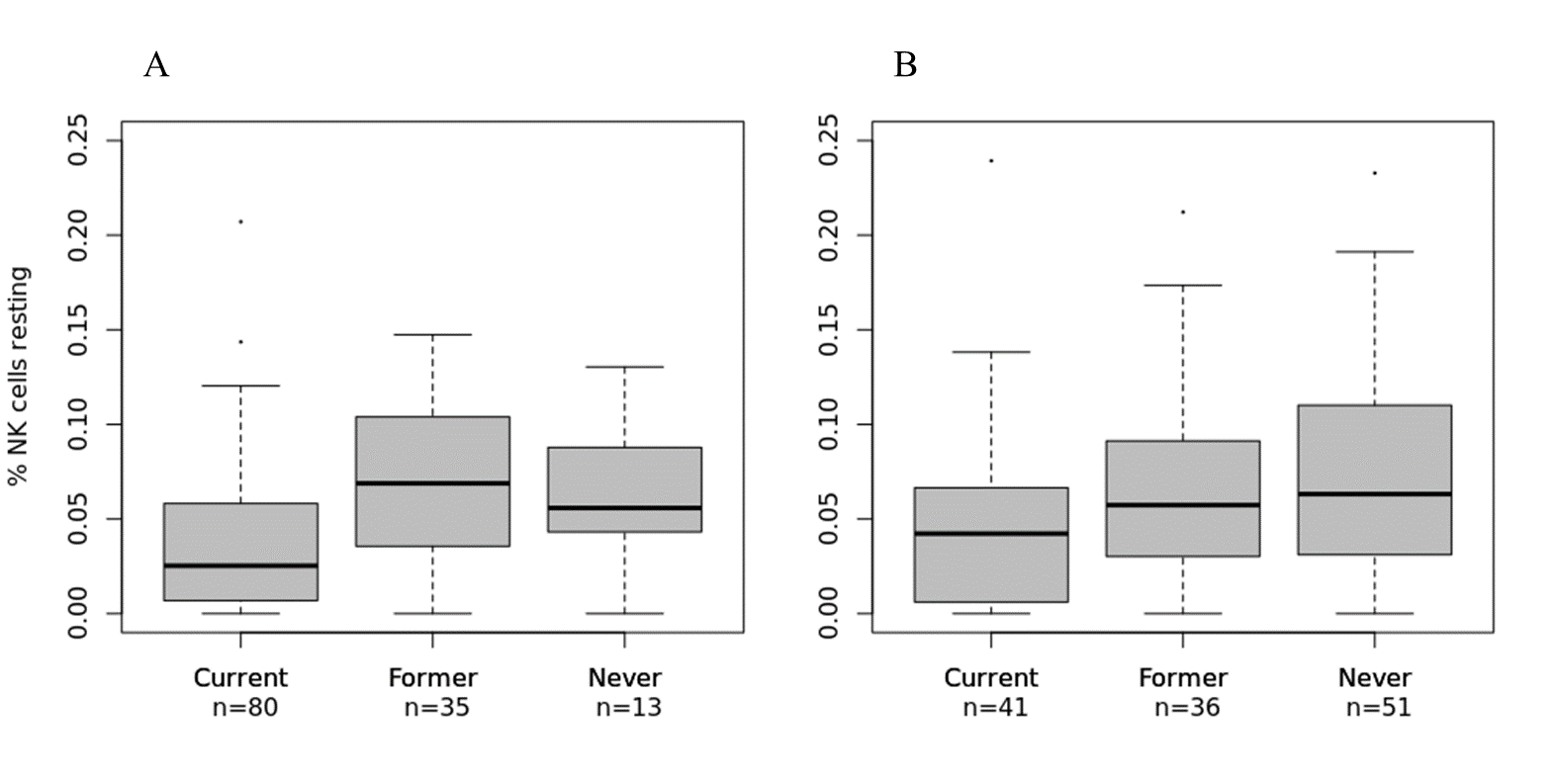
**

**Figure S2: Boxplot of estimated proportion of resting natural killer (NK) cells in blood samples obtained from cases (A, n=128) and controls (B, n=128) displayed according to smoking status.**

**
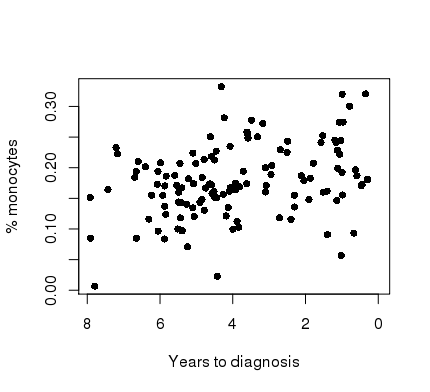
**

**Figure S3: Boxplot of estimated proportion of monocytes in blood samples from cases (n=128) according to the years between sampling and diagnosis.**

**Figure S4: Plots of p-values** **representing tests of whether the mean difference in log_2_ gene expression for case-control pairs = 0 (estimated from randomizing the case and control in each case-control pair) across days from blood sampling until time of diagnosis. Separate lines are presented for genes with order 50 (black), 200 (red), 500 (green), 1000 (blue), and 2000 (light blue), when ranked according to their difference within pairs. The p-value for time point t is equal to the p-value for the time period with middle point closest to t (after the p-values have been smoothed using a median filter with window size 99). The resulting curve is then smoothed using a mean filter with a window size of 1 month. The dotted horizontal line indicates a 0.05 level of significance, while the long vertical lines indicate the years before diagnosis.**

**
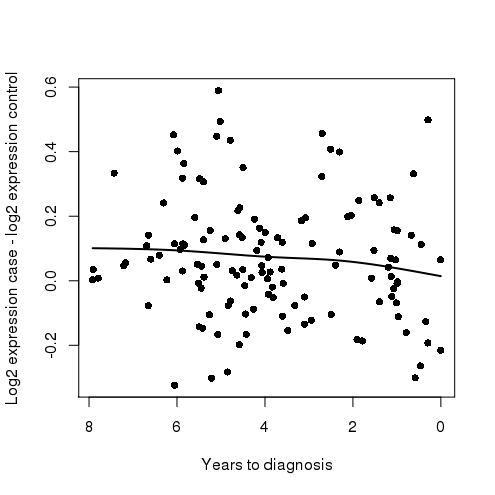
**

**Figure S5: The differences in log2 expression values for the gene *FGFR3* for cases and controls (n= 128 pairs) according to years to diagnosis based on spline regressions. The line represent a smoothed spline regression line.**
